# Organization of descending neurons in *Drosophila melanogaster*

**DOI:** 10.1038/srep20259

**Published:** 2016-02-03

**Authors:** Cynthia T. Hsu, Vikas Bhandawat

**Affiliations:** 1 Department of Biology, Duke University, Durham, North Carolina 27708, USA; 2 Deparment of Neurobiology, Duke University, Durham, North Carolina 27708, USA; 3 Duke Institute for Brain Sciences, Duke University, Durham, North Carolina 27708, USA.

## Abstract

Neural processing in the brain controls behavior through descending neurons (DNs) - neurons which carry signals from the brain to the spinal cord (or thoracic ganglia in insects). Because DNs arise from multiple circuits in the brain, the numerical simplicity and availability of genetic tools make *Drosophila* a tractable model for understanding descending motor control. As a first step towards a comprehensive study of descending motor control, here we estimate the number and distribution of DNs in the *Drosophila* brain. We labeled DNs by backfilling them with dextran dye applied to the neck connective and estimated that there are ~1100 DNs distributed in 6 clusters in *Drosophila*. To assess the distribution of DNs by neurotransmitters, we labeled DNs in flies in which neurons expressing the major neurotransmitters were also labeled. We found DNs belonging to every neurotransmitter class we tested: acetylcholine, GABA, glutamate, serotonin, dopamine and octopamine. Both the major excitatory neurotransmitter (acetylcholine) and the major inhibitory neurotransmitter (GABA) are employed equally; this stands in contrast to vertebrate DNs which are predominantly excitatory. By comparing the distribution of DNs in *Drosophila* to those reported previously in other insects, we conclude that the organization of DNs in insects is highly conserved.

A conserved feature of motor control across the animal kingdom is the anatomical separation between circuits that control rhythm generation, which are found in spinal cord/ventral nerve cord/segmental ganglia (collectively called body ganglia), and the circuits that integrate sensory information and initiate movement, which are found in the brain. These two circuits are connected by descending neurons (DNs), which have their cell bodies in the brain and carry sensory processing and motor-related information to the body ganglia, and ascending neurons (ANs), which have their cell bodies in the body ganglia and carry motor-related and sensory feedback information to the brain.

Because insects demonstrate highly differentiated motor repertoires while utilizing relatively few neurons, they are an excellent model system for unraveling the general principles of motor control. Individual DNs have been characterized in many species including cockroach (*Periplaneta americana*)^1^,^2^,^3^, cricket (*Gryllus bimaculatus*)^4^,^5^, moth (*Bombyx mori*)^6^,^7^, blowfly (*Calliphora erythrocephala*)^8^,^9^,^10^, locust (*Schistocerca gregaria*)^11^,^12^,^13^, and fruit fly (*Drosophila melanogaster*)^14^,^15^,^16^. Characterization of individual DNs provides much insight into descending motor control in insects. But to take advantage of the numerical simplicity of insects, a more comprehensive approach is necessary. Two studies have taken such an approach: One study in cricket^17^ and another in cockroach^18^ both used retrograde labeling from the cervical connective to quantify the number and distribution of DNs in cricket^17^. These studies showed that the DN population is similar in these two insect species and provided an anatomical framework for understanding information flow in the insect brain^18^.

Although the anatomical studies in cricket^17^ and cockroach^18^ have identified how DNs are organized, the lack of genetic tools in these insects have limited our progress in understanding descending motor control^19^. In contrast, these tools are readily available in *Drosophila melanogaster*. The availability of genetic tools as well as recent technical developments have made it possible to assess or manipulate the activities of identified neurons. This ability to probe identified neurons *in vivo* using either functional imaging^20^,^21^,^22^ or electrophysiological recording^23^,^24^ has led to a comprehensive understanding of the circuit basis of many computations in the brain. These include computations underlying sensory processing such as motion detection^25^,^26^ and olfactory processing^27^,^28^ and cognitive functions such as associative learning^29^ and spatial memory formation^30^.

In contrast to these advances in our understanding of sensory processing and decision-making in *Drosophila*, the neuronal circuit for descending control of movement remains understudied. Recent work has begun to characterize the role of individual DNs in specific tasks. For instance, recent studies have identified DNs that are crucial for backwards walking^14^, courtship song production^15^, and evasive take-off^16^. However, a comprehensive description of descending motor control is missing.

As a first step towards a comprehensive understanding of DNs in *Drosophila*, here we present an anatomical survey of DNs. We performed retrograde labeling of axons via the fly’s cervical connective to estimate the number and distribution of DNs. We also describe the projections of DNs, and the distribution of DNs according to expression of specific neurotransmitters. We find that the number and distribution of DNs is similar to that observed in other insect species, suggesting evolutionary conservation in the number and organization of DNs amongst insect species. Our study is the first (to our knowledge) to present the distribution of DNs in both the supraesophageal and subesopheageal zones, as well as the first study to present the organization of DNs according to the neurotransmitter they employ. We present our results in the context of studies of DNs in other insect species and in vertebrates.

## Results

Backfilling of the axons through the cervical connective labels a small number of somata in the brain in a distinctive pattern. These are the DN cell bodies (by definition). Backfilling also labels the neuropil in a characteristic pattern. Neuropil labeling reflects DN dendrites and axon collaterals, as well as the axons of ascending neurons (ANs). We first present our estimate of DN cell bodies and their distribution in the brain. Next, we describe the neural processes.

### *Drosophila* has ~1,100 DNs

The total number of cell bodies labeled in the brain varied between 837 and 907, with a mean of 878.5 DNs (±29.8 STD, n = 5) (Table 1). This variation likely reflects differences in the efficacy of the labeling procedure rather than an individual-to-individual variation in the number of DNs. Thus, higher numbers are likely to be closer to the actual number of DNs. Following the method of Okada and colleagues^18^, we also estimated the total number of DNs as a sum of the largest number of DNs observed for each cluster (see below) yielding an upper estimate of 1,113 DNs. Our method of backfilling will also label neck motor neurons that have their cell body in the brain as well as neurons that project through the cardiac recurrent nerve, resulting in a small overestimate for the number of DNs. In blowflies, it has been estimated that there are ~20 neck motor neurons^31^,^32^ and 16 neurons that project through the cardiac recurrent nerve^25^. Thus, these other populations make only a small contribution towards the overall cell count.

The narrow range of the numbers of labeled DNs across individuals and their stereotyped distribution suggests a high labeling efficiency. To estimate labeling efficiency, we performed retrograde labeling in flies in which a small number of DNs are genetically labeled. We chose *e49-Gal4/tsh-Gal80; UAS-mCD8-GFP/*+ flies because e49-Gal4 labels a small number of DNs in several DN clusters (see Supplementary Fig. S1 online). Because the genetically labeled DNs are isolated from other genetically labeled neurons in this line, we were able to unequivocally conclude that there are exactly 18 genetically labeled DNs in this line. We found that in the fly in which we labeled the largest fraction of DNs (out of 5 flies of this genotype that we bulk-labeled), we labeled 15 of the 18 genetically labeled DNs implying a labeling efficiency of 83%. In this same fly, a total of 883 DNs were labeled suggesting 1,060 DNs. This number is comparable to the number of DNs that we estimated by summing the maximum number per cluster.

The above experiment suggested a second method for measuring labeling efficiency: If the labeling efficiency were 100%, the number of genetically labeled axons in the cervical connective should be equal to the number of double-labeled cell bodies in the brain and thoracic ganglia. To facilitate axon counting, we employed tsh-Gal80 which represses Gal4 in most ANs, thus reducing the number of labeled axons in the cervical connective. By comparing the number of double-labeled neurons in the brain and thoracic ganglia to axons in the cervical connective, we were able to conclude that the labeling efficiency is high but we were not able to quantitatively assess the labeling efficiency (see Supplementary Fig. 1 for details).

The number of DNs reported in this study is remarkably similar to the numbers of DNs reported in previous studies performed in cockroach^18^ and cricket^17^. After accounting for the fact that the gnathal (formerly subesophageal) ganglia are not fused to the cerebral (formerly supraesophageal) ganglia in the cockroach and cricket as they are in *Drosophila*, the 235 pairs of DNs in cockroach and approximately 200 pairs of DNs in cricket match the 412 neurons (206 pairs) we found in the cerebral ganglia.

### DNs are organized in 6 clusters

**Figure 1:**
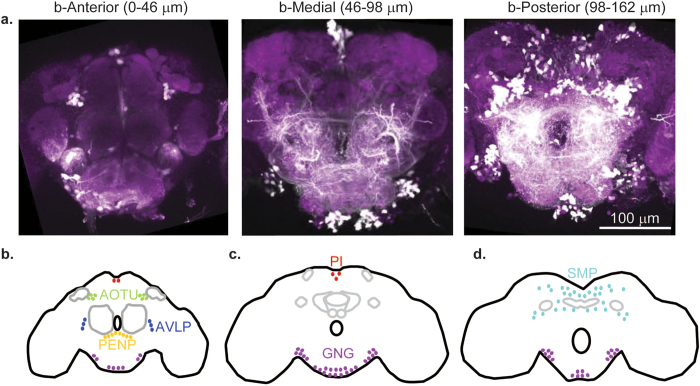
Drosophila has ~1,100 DNs distributed in 6 clusters. (**a**) Representative confocal image stacks showing the distribution of DN clusters. Maximum projection of dextran label (white) over the range of specified depths, superimposed on a section of neuropil containing the anatomical landmarks (anti-nc82, purple). (**b–d**) Schematic illustrating the mean distribution of DNs in the 6 clusters. Each cluster is represented with a different color. Each dot represents approximately 10 DNs.

In this section, we describe the distribution of DNs. Because the distribution of *Drosophila* DNs resembles the distribution of DNs in cricket^17^ and cockroach^18^, we will relate the distribution of *Drosophila* DNs to their descriptions in cricket^17^ and cockroach^18^. DNs have cell bodies distributed across 6 clusters (Fig. 1b–d). In the following section, we describe these clusters in order from their *b*-anterior to *b*-posterior positions in the brain, where the prefix “*b*-“ denotes the body axis rather than the embryonic neuroaxis (see methods and Ito *et al*.^33^ for more details). The number of neurons in each cluster, and the corresponding number of neurons in cricket and cockroach is reported in Table 1.

#### b-Anterior clusters

There are three anterior clusters: the anterior optic tubercle (AOTU) cluster, the anterior ventrolateral protocerebrum (AVLP) cluster, and the periesophageal (PENP) cluster (Fig. 1b,c). All three clusters contain relatively few DNs. The AOTU clusters and the AVLP clusters are paired (one in each hemisphere).

The AOTU cluster (Fig. 1b, olive green) is located lateral to the vertical lobe of the mushroom body and medial to the anterior optic tubercle. Neurons in this cluster send projections through the medial antennal lobe tract (mALT) toward the ventromedial neuropils. Based on both the soma location and the projection of the primary neurites, this cluster corresponds to the cluster i5 described in cricket^17^ and cockroach^18^. In the cricket, an additional cluster was identified, i5n, which was medial to cluster i5 and whose neurites projected along a distinct but parallel tract. Although we could not distinguish two distinct clusters of soma in our study, two sets of neurites projecting from this cluster are labeled (data not shown), suggesting that the AOTU cluster in *Drosophila* may also be subdivided into two separate clusters.

The AVLP cluster (Fig. 1b, dark blue) is located between the anterior ventrolateral protocerebrum and the antennal lobe. The AVLP cluster does not have an obvious equivalent in cockroach^18^, but may be analogous to cluster i6 in the cricket^17^.

The PENP cluster (Fig. 1b, golden yellow) is located between the prow of the periesophageal neuropil and the antennal lobe. The location of this cluster corresponds to cluster i7 previously reported in the cockroach and cricket^17^,^18^.

#### Pars Intercerebralis (PI)

The PI cluster (Fig. 1c, red) is located between the hemispheres of the superior medial protocerebrum. Neurons in this cluster send projections into the median bundle^34^. Of these neurons, 14 have been characterized in *Drosophila* as insulin producing cells (IPCs) that project through the cardiac recurrent nerve to the corpora cardiac and the associated aorta, proventriculus, and crop, rather than through the ventral nerve cord to the thoracic ganglia^34^,^35^. However, a previous study in the locust has identified at least one DN from the PI cluster which innervates the thoracic ganglia^11^. In addition, studies in the cockroach have also identified DNs with soma located in the PI^2^.

#### Gnathal Ganglia (GNG)

The GNG cluster is the largest DN cluster, containing an average of 483.2 neurons (±61 STD, n = 5) (Fig. 1b–d, lavender). This cluster is further subdivided into a medial cluster and two lateral clusters. The medial cluster is found near the *b*-ventral (*n*-posterior) surface of the gnathal ganglia, while the lateral clusters are found lateral to the gnathal ganglia and *b*-ventral (*n*-posterior) to the saddle. There were 187.8 neurons (±62.9 STD, n = 5) in the medial GNG cluster and 110.5 neurons (±33.5 STD, n = 10) in each of the lateral GNG clusters. Because of ambiguity in assigning neurons to medial versus lateral clusters, in Table 1 we report these clusters as a single cluster. The number of GNG-DNs we report here is similar to a previous study in the locust^36^, which reported a total of 153 neurons labeled through introduction of cobalt chloride into one of the two cervical connectives.

#### Superior Medial Protocerebrum (SMP)

The SMP cluster consists of cell bodies distributed in the posterior superior medial protocerebrum, the superior intermediate protocerebrum, the posterior lateral protocerebrum, the inferior bridge, and the inferior and superior clamp (Fig. 1d, cyan). In the cricket and cockroach studies, the fact that the authors only labeled one connective allowed the authors to divide this cluster into four clusters – i1(or c1) through i4 (or c4), where “i” vs “c” refer to whether the neurons project to the ipsilateral or contralateral side of the brain^17^,^18^. The cricket and cockroach studies also described a large soma projecting into the ocellar tract that separated clusters i3 and c3 from i4 and c4 clusters. However, in *Drosophila*, unlike in the cockroach, the ocellar nerve is found in the midline of the brain^3^,^37^,^38^; thus the ocellar nerve was not a feasible landmark. Because of the lack of clear landmarks and thus a lack of obvious boundaries between the four clusters, in this study we report the number of neurons in the four clusters as a single number.

### Neuropil labeled by backfill from cervical connective

**Figure 2:**
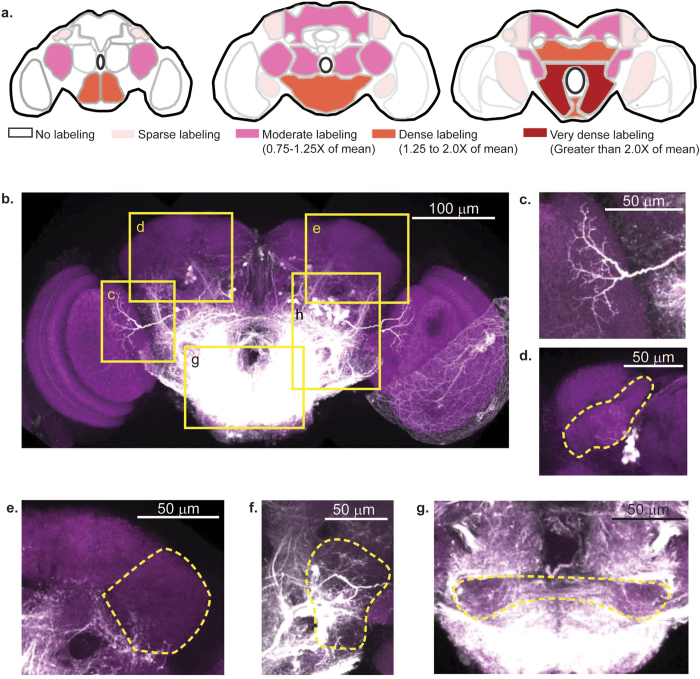
Pattern of neuropil labeling suggest distinct regions for sensory, associative, and motor processing. (**a**) Density of labeling for different neuropil regions. (**b**) Projections of a confocal stack show dextran labeling (white) and neuropil labeling (magenta). Regions marked in yellow are expanded in panels (**c–g**) to show sparse innervation of different brain regions. (**c**) Sparse innervation of the lobula (**d**) Sparse innervation of the anterior optic tubercle. **(e)** Sparse innervation of the lateral horn. (**f**) Dense innervation of the posterior ventrolateral protocerebrum, where optic glomeruli are found. (**g**) Dense innervation of AMMC and surrounding neuropil. For clarity, only a single representative 1 μm slice is shown. The extent of the image stack is different for the images in (**b–g**).

In addition to the cell bodies, backfill through the neck also labels the axons and dendrites of DNs and axons of ANs. The pattern of neuropil labeling was distinct and consistent from brain to brain. We could not distinguish between DN and AN processes and therefore report the overall neuropil labeling. The density of labeling for different neuropil regions (averaged over 5 brains) is shown in Fig. 2a.

The labeling was densest in the posterior slope, located in the *b*-posterior region of the brain. In contrast, known associative areas of the brain—the mushroom body and the central complex—had no detectable labeling, suggesting that these regions neither send direct outputs to nor receive direct inputs from the thoracic ganglia.

Some sensory neuropils were sparsely labeled: these include the lobula (Fig. 2c), the optic tubercle (Fig. 2d), and the lateral horn (Fig. 2e). This sparse labeling of sensory neuropil suggests that these regions are innervated by very few DNs (or ANs), and that most sensory information represented in these brain regions is further processed before being relayed to the DNs. In contrast, the ventrolateral protocerebrum, the majority of whose volume is composed of optic glomeruli that receive output from optic lobes, is densely labeled^39^,^40^ (Fig. 2f). Previous studies in both *Drosophila*^41^ and *Calliphora*^42^ have also identified individual DNs whose dendrites innervate the optic glomeruli. The AMMC (antennae mechanosensory and motor center, Fig. 2g), which processes mechanosensory information, is also densely labeled. This is in agreement with previous studies which have identified descending neurons that carry information from the AMMC to the thoracic ganglia^41^,^43^.

The distribution of neuropil labeling is consistent with that observed in the cockroach^18^ and suggests that, as in the cockroach, both direct and indirect pathways connect cephalic sensory processing to behavior^18^ in *Drosophila*. Thus, information from AMMC and optic glomeruli can be directly communicated to DNs. At the same time, a lack of labeling in mushroom body and central complex implies that processing in these centers affect behavior indirectly; in this case the effect of sensory input on DNs is separated by at least two synapses.

### Distribution of DNs by neurotransmitter

**Figure 3:**
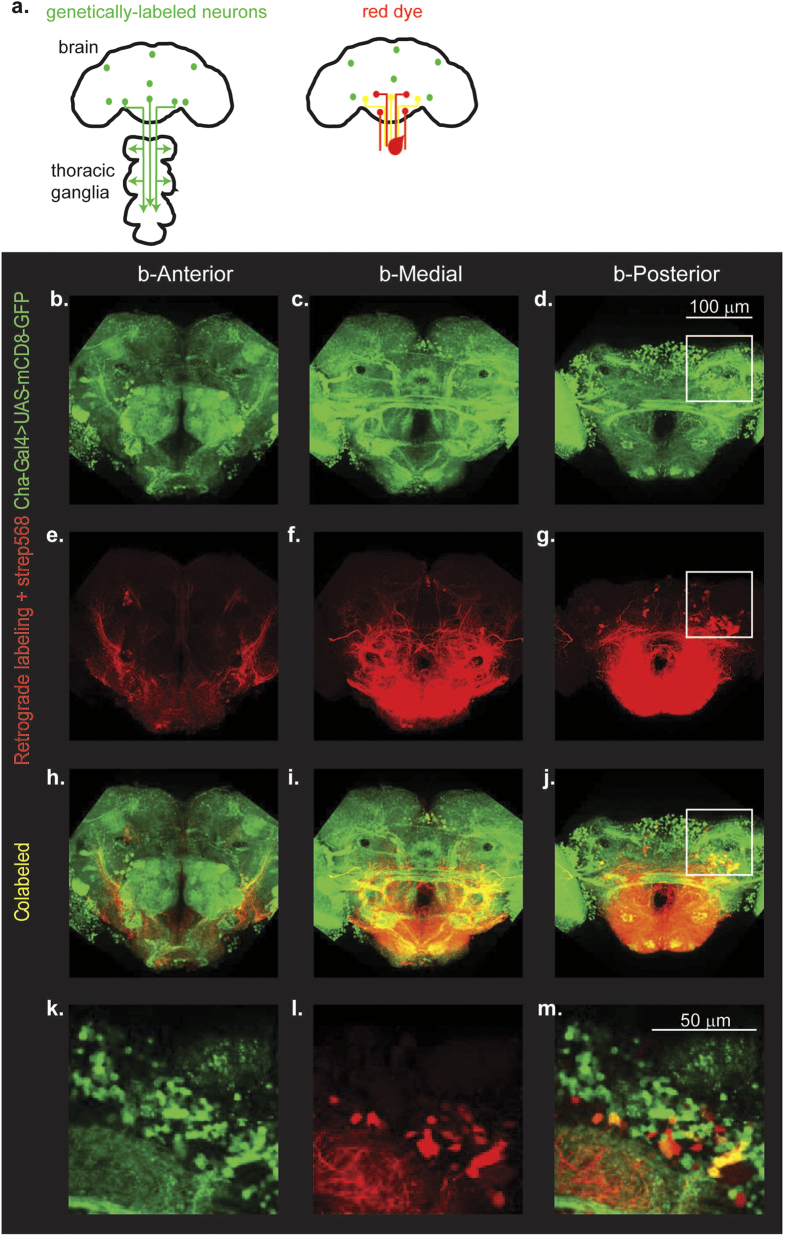
Strategy for labeling DNs with a given neurotransmitter. (**a**) Schematic illustrating that the subset of GFP+ neurons labeled by a Gal4 driver can be identified as DNs if they are also colabeled by retrograde labeling (yellow). (**b–j**) Projection of a confocal stack of a retrogradely labeled brain in which all cholinergic neurons are labeled using Cha-Gal4,UAS-GFP (green). Retrograde label is in red. Cholinergic DNs are colabled and appear yellow. (**k–m**) Close-up of the region in white square in (**d,g,j**) show the co-labeled neurons.

Because the neurotransmitter used by a given neuron is an important determinant of its function, we wanted to estimate the distribution of DNs by neurotransmitter. To label all DNs that utilize a given neurotransmitter, we perform labeling in flies in which the neurons utilizing a given neurotransmitter is also labeled (Fig. 3a). For instance, Fig. 3b–d shows projections of the anterior, medial, and posterior portions of the brain in a Cha-Gal4, UAS-GFP fly. Multiple studies have shown that Cha-Gal4 labels most cholinergic neurons^44^,^45^. When this is combined with red dextran dye (Fig. 3e–g), the neurons that are labeled with both GFP and red dextran dye appear yellow (Fig. 3h–j,m). These neurons are the DNs that express the neurotransmitter in question (in this case cholinergic DNs).

**Figure 4:**
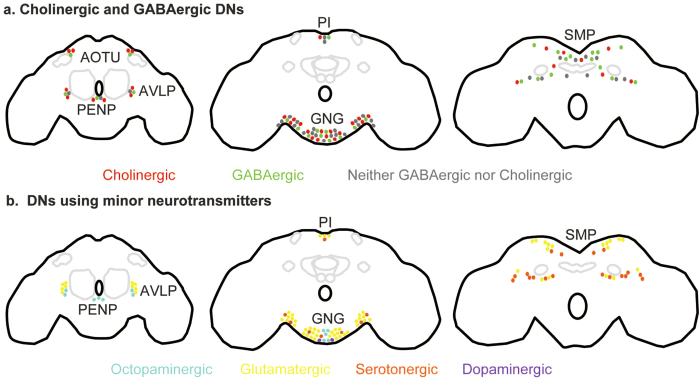
Schematics showing distribution of DNs by neurotransmitter. (**a**) Distribution of cholinergic (red) and GABAergic DNs (green). Dots are in proportion to the fraction of DN of a given type. (**b**) Distribution of DNs which employ other (minor) neurotransmitters. Each dot is a single DN.

We performed experiments analogous to the ones described in Fig. 3 for acetylcholine (n = 4), GABA (n = 4), glutamate (n = 4), octopamine (n = 4), and serotonin and dopamine (n = 4). We found that DNs as a population use all the neurotransmitters we tested (Table 2). As in the counts for total number of neurons, we assumed that variability in the number of DNs reflected variability in labeling efficiency; therefore, we report the maximum number of DNs per cluster rather than the mean. We first describe the distribution of DNs that belong to the major excitatory and inhibitory neurotransmitter types followed by DNs which do not use these major neurotransmitters (Fig. 4, Table 2 and Supplementary Table S1).

#### Major neurotransmitters: Cholinergic and GABAergic DNs

The major excitatory neurotransmitter in the *Drosophila* brain is acetylcholine^45^,^46^. To label cholinergic neurons, we performed bulk-labeling in *Cha-Gal4, UAS-GFP* flies (Figs 3b–m and 4a). To label DNs which express the major inhibitory neurotransmitter, GABA^47^,^48^,^49^, we employed two methods: First, we performed experiments in *Gad1-Gal4;UAS-mCD8-GFP* flies. Second, we performed labeling in a standard lab strain (w^1118^) and used an anti-GABA antibody to label GABAergic neurons. We found that for all clusters except the GNG cluster, more neurons were labeled by the antibody method than by Gad1-Gal4. This is consistent with previous reports which show that Gad1-Gal4 does not label all GABAergic neurons^50^. Therefore, we report our results with the GABA antibody (Fig. 4a, Table 2, and Supplementary Fig. S2 and Table S1).

We found that all clusters contain both cholinergic and GABAergic DNs (Fig. 4a, Table 2, and Supplementary Table S1). However, the majority of the DNs in two of the anterior clusters (AOTU and PENP) were cholinergic. A greater fraction of the SMP DNs was GABAergic, while a greater fraction of the GNG DNs was cholinergic (especially in the lateral GNG). Overall roughly 40% of the DNs are cholinergic and 40% are GABAergic.

#### Minor neurotransmitters

There are 56 glutamatergic DNs (Fig. 4b and Supplementary Fig. S3). Glutamatergic neurons are labeled in *Vglut-Gal4;UAS-CD8GFP* flies, in which Gal4 is driven under the control of the vesicular glutamate transporter (Vglut). These are distributed in all clusters except for the AOTU and the PENP cluster, but most are found in the SMP and the GNG clusters (9 and 18 DNs, respectively, per hemisphere). Of these, at least 3 pairs of DNs in the SMP cluster and 5 pairs of DNs in the GNG cluster may be neck motor neurons, assuming homology between the blowfly *Calliphora* and *Drosophila*^31^. Consistent with this study, a previous study in the honeybee also found 5 DNs with glutamatergic-like reactivity located in the ocellar tract (the midline of the SMP cluster) and 16 additional DNs elsewhere in the SMP^51^.

The next most common neurotransmitter used is serotonergic (Fig. 4b and Supplementary Fig. S4). Serotonergic DNs were labeled using *Ddc-Gal4* which labels both dopaminergic and serotonergic neurons and labels up to 30 DNs (summed over all clusters). Out of these, 2 DNs located in the GNG have been confirmed to be dopaminergic by performing labeling in TH-Gal4 flies in which only dopaminergic neurons are labeled^52^. Eighteen of the serotonergic DNs are in the SMP cluster and occur in a location similar to serotonergic DNs reported in locust^53^. The other 10 are present in the GNG cluster. Two of the lateral GNG serotonergic DNs have been previously reported in the moth (*Manduca sexta*) and described as also innervating the labial neuromere of the GNG^54^. There are also reports of a pair of medial GNG serotonergic DNs in the blowfly (*Calliphora*)^53^.

Tdc2-Gal4, which labels octopaminergic neurons, labels 12 DNs (Fig. 4b and Supplementary Fig. S5). Octopaminergic DNs are distributed across three clusters (PENP, AVLP, and GNG). Ten of these DNs have been described previously^55^.

Assuming that most DNs only employ a single neurotransmitter, 85% of the DNs can be attributed to utilizing one of the five neurotransmitters we characterized. Some of the remaining 15% of DNs represent small diameter DNs which use minor neurotransmitter and are not labeled. We think that this is unlikely because we have shown in a previous study that small diameter dopaminergic neurons are reliably labeled^52^. Moreover, all the octopaminergic DNs that have been labeled genetically^55^ were also identified in our study. Therefore, it is more likely that the remaining 15% of DNs use neurotransmitters not characterized in this study: histamine, tyramine or peptidergic neurotransmitters. For instance, previous studies in the moth *Manduca sexta* have found DNs that express FMRFamide in the midline of the GNG^56^. Our strategy for labeling DNs also label neurosecretory cells, such as those found in the PI and in the pars lateralis, whose location coincides with the SMP cluster. These neurosecretory cells secrete neuropeptides such as *Drosophila* insulin-like peptide and FMRF, and thus they may not express any of the neurotransmitters examined in this study^35^,^57^.

## Discussion

We present here, what is to our knowledge, the first comprehensive description of the number and distribution of DNs in *Drosophila*. In the following, we discuss the organization of DNs in the context of studies that describe the organization of DNs in other invertebrates and vertebrates.

Our results suggest that *Drosophila* have ~1,100 DNs that are distributed across 6 clusters. Using a genetic strain in which a small number of DNs were labeled (Supplementary Fig. S1), we were able to show that the labeling efficiency is high. Similarly, the number and distribution of octopaminergic DNs in our study matches the description of octopaminergic DNs^55^ in another study that employs a completely different approach. Thus, our estimate of ~1,100 DNs is likely to be close to the actual number of DNs. Previous studies have also shown that there are ~3,600 axons which traverse the cervical connective in *Drosophila*^58^. Based on our work we estimate ~1,100 of these are descending axons, while ~2,500 are ascending axons that represent a combination of ascending motor and sensory input into the brain from the thoracic ganglia.

Our results also show that in *Drosophila*, DNs employ multiple neurotransmitters and no DN cluster exclusively expresses any single neurotransmitter type. A hallmark of descending motor control is that, irrespective of size and complexity of the movements being controlled, descending control systems employ DNs of multiple neurotransmitter type^59^; the use of multiple neurotransmitter type is considered important for flexible control of behavior. Thus, it is not surprising that the use of multiple neurotransmitter types by the DN population is also observed in the fly. In this study, we provide the number and location of neurons which employ each of the different neurotransmitters. This will facilitate our understanding of how different neuromodulatory DNs affect a fly’s motor behavior.

**Figure 5:**
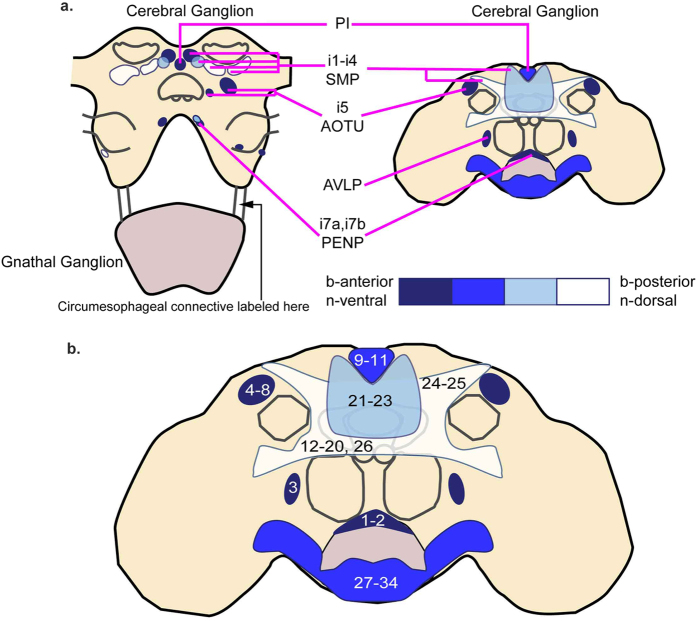
Schematics showing that the organization of DNs is conserved across insects. (**a**) Distribution of DN clusters in hemimetabolous insects (cricket^17^, cockroach^18^) relative to landmark neuropil regions (antennae lobe, central body, mushroom body calx). This is shown in comparison to the distribution of DN clusters in *Drosophila* (right), a holometabolous insect, as described in this study. The gnathal ganglion, which in holometabolous insects such as *Drosophila* is fused to the cerebral ganglia, is shown shaded in lavendar. (**b**) The approximate locations of insect DNs previously described in the literature is shown in the context of the clusters described in this study. The numbers correspond to rows listed in Table 3.

A comparison of our study to previous studies suggests a high degree of conservation in the number and organization of DNs across arthropods. The number of DNs in the cerebral ganglia of flies is similar to the number reported in cricket^17^ and cockroach^18^, while the number of DN cell bodies we found in the gnathal ganglia is similar to the number previously described in locusts^36^. There is also a clear homology (see Fig. 5a) in the spatial distribution of DNs in the brain. The similarity between cricket^17^, cockroach^18^ and fly (this study) is also supported by a study in another holometabolous insect, the moth *Bombyx mori*, which found three different groups of DNs in the cerebral ganglia^60^, corresponding to the AOTU, SMP, and PENP clusters described in this study. Additionally, we reviewed most (if not all) reports of DNs in insects and were able to assign them to one of the clusters (Fig. 5b and Table 3).

Given the overall conservation of the structure and function in the arthropod brain^33^ and the reports of many homologous neurons such as the giant fiber neurons and the neck motor neurons^61^, the conservation in the distribution of DN clusters across insect orders is not surprising. But the remarkable conservation in the number of DNs is surprising given that the number of neurons in other structures such as antennal lobe^62^, optic lobe^62^ and mushroom body^63^ varies by several-orders of magnitude across different insect species. This conservation might reflect the fact that the number of muscles is similar across insects. Since most insect muscles are innervated by 1 to 3 motor neurons (no more than 13 motor neurons)^64^, insect motor systems likely have similar level of complexity. A similar result was also observed in comparing analogous brain regions in bumblebees to honeybees^65^. The authors found that sensory areas of the brain and mushroom body scaled with the size of the insect, but the central body (also referred to as central complex), which is associated with movement control, was smaller relative to brain size in the larger insects.

Cricket (order: orthoptera) and cockroach (order: Blattodea) are both hemimatabolous insects, while *Drosophila* is a holometabolous insect^33^. Since hemi- and holo- metabolous insects diverged at least 280 million years ago^33^,^66^,^67^, the similarities in the number and distribution of DNs among these insects implies a high level of conservation. In addition to the conservation in DN numbers across the insect class, the number of supraesophageal DNs in lobster have been estimated to be around 600–700^68^, suggesting that DN numbers are conserved across arthropods.

The organization of DN processes is also evolutionarily conserved among insects. Although we were not able to distinguish between axonal and dendritic processes, the overall organization of the labeled neural processes is strikingly similar to the cockroach study^18^. Both our study and the cockroach study suggest that DNs receive input from regions of the brain that are innervated by outputs from mushroom body and central complex. Equally importantly, neither DNs nor ANs innervate the central complex or mushroom body, implying that these neuropils do not directly affect motor output. Thus, one important pathway in the insect brain for information flow from sensory circuits to motor circuits is through the mushroom body and central complex to the DNs. There is also direct sensory input into descending neurons (DNs): The labeling presented in this study is consistent with visual inputs into DNs from optic glomeruli and mechanosensory input from AMMC. Similarly, the large number of DNs and dense labeling in the GNG is consistent with the prominent role of GNG in motor control.

In contrast, our study raises three important differences between descending motor control in vertebrates versus arthropods. The number of DNs in arthropods is 3-orders of magnitude smaller than in the vertebrates, which often possess upward of a million DNs^69^,^70^. This difference is unlikely to be due to the difference in the number of muscles because the number of muscles is surprisingly similar between insects and mammals^62^: the 296 skeletal muscles possessed by locust is comparable to the 316 muscles present in some primates and exceeds the number of muscles in rodents^71^. The difference between the number of DNs in the vertebrate and invertebrate model systems likely originates in the differences in the neuronal control of muscles^72^,^73^. A vertebrate skeletal muscle can be innervated by hundreds of motor neurons^64^. The dominant mechanism for regulating the amount of force generated by a vertebrate muscle is through the sequential recruitment of motor units. In contrast, arthropod muscles are typically innervated by only 1 to 3 motor neurons and there are rarely more than 13 motor neurons innervating a given muscle^64^. Arthropod muscles produce a gradation of force by activating individual muscles in a graded fashion. Because of the differences in the neural control strategies of muscle contraction, the total number of motor neurons in vertebrates is at least three-order of magnitude greater than in invertebrate systems: There are ~50,000 motor neurons^74^ innervating limbs in a human compared to ~50 in Drosophila^75^. In comparison to this three-orders of magnitude difference in the number of motor neurons, the ratio of interneurons in the body ganglia to motor neurons only scales modestly: the ratio is 25:1 for macaque^76^, 14:1 for mouse and 8:1 for turtle^77^; a similar ratio of 10:1 has been reported for locusts^78^. Thus, it is plausible that the large difference in DN numbers between vertebrates and invertebrates arise from the differences in the number of motor neurons.

Second, comparative study of DNs in vertebrates has revealed that several descending tracts such as reticulospinal tracts and vestibulospinal tract are common to all vertebrates^79^. Others have evolved during the course of vertebrate evolution. There is also a great variation in the relative number of neurons in each descending tract in different species^69^,^79^. These differences in the organization of vertebrate DNs contrast with the relatively conserved DN numbers in arthropods. Thus, the evolution of DNs within the vertebrate phyla and invertebrate phyla seems to follow a fundamentally different plan.

Finally, the two major clusters of DNs in flies, SMP and GNG, contain comparable numbers of excitatory cholinergic neurons and inhibitory GABAergic neurons. This contrasts with the largely excitatory neurons which comprise DNs in mammals. The corticospinal tract, rubrospinal tract and the vestibulospinal tract are all glutamatergic and thus presumably excitatory^80^. Only the reticulospinal tract has a significant fraction of GABAergic neurons; although, even in this case the 59% glutamatergic neurons dominate the 20% GABAergic axons^80^. This difference in neurotransmitter might reflect a fundamentally different logic underlying descending control in these two phyla.

These differences do not imply that vertebrate and invertebrate motor systems are fundamentally different. Both motor systems control rigid, articulated skeletal system using muscles which are in turn controlled by neurons. The architecture underlying neural control of movement is similar in both systems and involves descending control of central pattern generators. With respect to DN pathways, there are many functional parallels. Both the vertebrate brainstem and the gnathal ganglia DNs contain tonically firing neurons which are strongly activated during locomotion^52^,^81^. They also both play a crucial role in regulating indirect aspects of motor control such as respiration and posture and interact with other tracts in the brain in a parallel, hierarchical, but recurrent fashion^81^. Similarly, functions subserved by DNs in the cerebral ganglia parallel functions carried out by DNs which originate from the cortex or red nucleus in mammals. Many previously characterized sensory specific DNs in insects also have cell bodies located in the cerebral ganglia, such as the descending neurons of the ocellar and vertical system (DNOVs)^82^, the descending contralateral motion detector (DCMD)^13^, and the target selective descending neurons (TSDNs)^83^. In particular, the TSDNs operate similarly to the DNs in the vertebrate corticospinal tract: they process behaviorally relevant sensory information (visual stimuli resembling prey) and relay information directly to the wing motor centers using a population vector representation. Taken together, there are many features of descending motor control in invertebrate systems that suggest that they operate on principles similar to that in vertebrates. Thus, a detailed description of the DN population and its distribution in different parts of the brain in the *Drosophila* will inform future models of how different descending tracts from the brain cooperate to control movement.

## Methods

### Fly stocks

Flies were reared on standard cornmeal-agar and raised at 25 °C. In all experiments, adult female flies at least 18 hours post-eclosion were used. Fly stocks were obtained from the Bloomington Stock Center, with the exception of E49-Gal4^84^ (a gift from Kristin Scott).

The neurotransmitter used by DNs was inferred from double labeling by both the dextran and a label for the neurotransmitter. To label GABAergic neurons, we used anti-GABA antibody. The genotype of the flies used to label the other neurotransmitters employed by different DNs are as follows: for cholinergic DNs, the genotype was *Cha-Gal4,UAS- GFP*; for serotonergic DNs, the genotype was *Ddc-Gal4/*+*; UAS-mCD8-GFP/*+; for octopaminergic neurons, the genotype was *Tdc2-Gal4/UAS-mCD8-GFP; UAS-mCD8-GFP/*+; for glutamatergic neurons, *VGlut-Gal4/VGlut-Gal4; UAS-mCD8-GFP/UAS-mCD8-GFP*.

### Retrograde labeling of DNs

A small well was dotted with a very small amount of vacuum grease (Dow Corning) to stabilize the severed head of a fly. Heads were placed antennae-side down. Biotinylated 3K tetramethylrhodamine dextran (Invitrogen D7162) was applied to the severed neck connective. Dextran was prepared as a 10% w/v solution (10g in 100 ml) in phosphate buffered saline (PBS); 0.5 μl of this solution was used to label each brain. Fly heads were then immersed in *Drosophila* external saline^85^ (in mM: 103 NaCl, 5 KCl, 5 Tris, 10 glucose, 26 NaHC03, 1 NaH2P04, 1.5 CaCl2, 4 MgCl2, osmolarity adjusted to 270–285 mOsm, and bubbled with 95% O2/5% C02 to pH 7.1–7.4) and left at room temperature for 30–90 minutes. Brains were then dissected in PBS and fixed for 20 minutes in a 4% paraformaldehyde solution (in PBS). The brains were then stained using the immunocytochemistry protocol described below. Confocal fluorescence microscopy was performed using a Zeiss 510 upright confocal microscope. Confocal Z-stacks were acquired at 1 μm interval using a 40X objective. Images were stitched together using the Pairwise Stitching PlugIn developed for ImageJ^86^.

### Immunocytochemistry

After retrograde labeling, the fixed brains were processed using a standard immunocytochemistry protocol: Fixed brains were blocked for 20 minutes in a solution of 5% normal goat serum (NGS) and 0.4% PBST. The brains were incubated with primary antibodies in 5% NGS blocking solution at 4 degrees for 1–3 days. Brains were next rinsed 3 × 10 minutes in PBST and placed in secondary antibodies in 5% NGS blocking solution at 4 degrees for 1–3 days. Finally, brains were rinsed 3 × 10 minutes in PBST and mounted in Vectashield (Vector Labs) for confocal imaging. Primary antibodies used were mouse anti-nc82 (1:40, DSHB), rat anti-mCD8 (1:40, Invitrogen MCD07800), rabbit anti-GFP (1:500, Invitrogen A11122), and rabbit anti-GABA (1:100, #A2052; Sigma, St. Louis, MO). Secondary antibodies used were goat anti-mouse 633 (1:400, Invitrogen A21050), goat anti-rat 488 (1:400, Invitrogen A11006), goat anti-rabbit 488 (1:500 when amplifying anti-GFP, 1:250 when amplifying anti-GABA; Invitrogen A11034) and streptavidin 568 (1:500, Invitrogen S11226).

### Nomenclature and terminology

We have adhered to the nomenclature recommended by the consortium “The Insect Brain Name Working Group”^33^. Because published work (Ref. 20) describes this nomenclature in details, we describe our naming conventions in brief. Consistent with the conventions suggested by the working group, the location of cell bodies is described with respect to the body axis (specified with a prefix b-). However, to maintain consistency with previous studies in other insects, which use the embryonic neuroaxis, we also specify the neuroaxis and add the prefix “n-” (as opposed to “b-”) when relevant.

To describe major brain regions, we used neuromere based definitions (cerebral ganglia and gnathal ganglia) rather than esophagus-based definition (supraesophageal and subesophageal), to facilitate comparison of the anatomy between holometabolous insects such as *Drosophila* and the hemimetabolous insects, such as the cockroach and the cricket. The cerebral ganglia consist of the protocerebrum, deutocerebrum and tritocerebrum while the gnathal ganglia consists of the maxillary, mandibular and labial neuromeres. In holometabolous insects, such as *Drosophila*, the deutocerebrum and tritocerebrum are located in the subesophageal zone, while in the hemimetabolous insects, they are located in the supraesophageal zone.

DN clusters were named according to the neuropil adjacent to them. The consortium study describes the major neuropil as Level 1 neuropil which is then further subdivided into Level 2 neuropil. Three of the six clusters of DNs were named according to the Level 2 neuropil they were found adjacent to: anterior optic tubercle (AOTU), anterior ventrolateral protocerebrum (AVLP), superior medial protocerebrum (SMP). The gnathal ganglion (GNG) cluster was named for the Level 1 neuropil because no Level 2 divisions have been described yet. The periesophageal neuropil cluster (PENP) was named for the Level 1 neuropil rather than the three structures it was adjacent to because of the difficulty in distinguishing the Level 2 structures (specifically the flange, the prow, and the cantle). The remaining cluster of neurons, the Pars Intercerebralis (PI) cluster, has been previously described in the literature, and has not been reassigned a name by the Insect Brain Name Working Group.

### Analysis of Density of Labeling

To quantify the intensity of neuropil labeled by dextran, the confocal stack was sampled at 10 μm intervals. The neuropil was manually divided into regions of interest on each slice of the 10 μm substack. The mean intensity of all the pixels within each region indicated on each slice of the 10 μm substack was computed, resulting in a single mean intensity for the volume of interest. The neuropils quantified were those listed as Level 2 neuropils in Ref 20, except for the inferior neuropils, mushroom body, central complex, lateral complex, and periesophageal neuropils, which were consolidated according to their Level 1 supercategories for convenience.

## Additional Information

**How to cite this article**: Hsu, C. T. and Bhandawat, V. Organization of descending neurons in *Drosophila* *melanogaster*. *Sci. Rep*. **6**, 20259; doi: 10.1038/srep20259 (2016).

## Supplementary Material

Supplementary Information

## Figures and Tables

**Table 1:** Number of DNs per cluster.

This study				Corresponding Cluster in cricket and cockroach	Cricket^17^**		Cockroach^18^**	
Cluster Name	Mean ± SD	Median	Max		Median	Max	Median	Max
AOTU (n = 10*)	17 ± 17.1	19	38	i5,i5n	10	22	23	35
AVLP (n = 10*)	23.5 ± 23.7	18	29	NA	NA	NA	NA	NA
PENP (n = 5)	34.4 ± 23.7	18	80	i7a,i7b,c7	6	17	11	18
PI (n = 5)	34.3 ± 12.5	26	48	PI	5	19	2	6
SMP(n = 5)	277.5 ± 48.4	280	325	i1-i4,c1-c4	111	154	116	169
GNG(n = 5)	483.2 ± 38.3	462	526	NA	NA	NA	NA	NA

^*^For AOTU and AVLP clusters, n refers to the number of hemispheres rather than the number of flies.

^**^Counts for cricket and cockroach represent pairs of neurons (only one connective was labeled in those experiments).

**Table 2:** Percent DNs expressing each neurotransmitter.

Neurotransmitter	Labeled using	Percent
Acetylcholine	Cha-Gal4 > GFP (neurons expressing choline acetyltransferase)	38
GABA	GABA antibody	37
Glutamate	VGlut-Gal4 > UAS-mCD8-GFP (neurons expressing vesicular glutamate transporter)	6*
Serotonin	Ddc-Gal4 > UAS-mCD8-GFP (neurons expressing dopamine decarboxylase)	3*
Octopamine	Tdc2-Gal4 > UAS-mCD8-GFP (neurons expressing tyramine decarboxylase)	1*
Dopamine	TH antibody	0.2*
Total		85

For acetylcholine and GABA, the percentage was computed by summing the largest number of colabeled neurons in each cluster, then dividing by the total number of bulk labeled neurons found in those clusters (see Supplementary Data for details).

^*^Percent for neuromodulators reported as the result of dividing the sum of the maximum number of colabeled neurons per cluster by 900, the average number of neurons labeled by our bulk labeling technique.

**Table 3:** DNs characterized in insects.

	Neuron	Species
PENP		
1	Group I flip flopping DNs^6^,^7^,^60^,^87^	*Bombyx mori*
2	Fru + aDT8^88^	*Drosophila melanogaster*
AVLP		
3	Octopaminergic neurons (OA-VL1, OA-VL2)^55^	*Drosophila melanogaster*
AOTU		
4	Group II flip flopping DNs^6^,^7^,^60^,^87^	*Bombyx mori*
5	DBNi5-1-19^4^	*Gryllus bimaculatus*
6	DBNi5n-1^4^	*Gryllus bimaculatus*
7	DBNc5-4^4^	*Gryllus bimaculatus*
8	DBNc5-5^4^	*Gryllus bimaculatus*
PI		
9	Polarization sensitive contra DN^11^	*Schistocerca gregaria*
10	D3, D4^2^	*Periplaneta americana*
11	D5^2^	*Periplaneta americana*
SMP		
12	DBNi1-3^4^	*Gryllus bimaculatus*
13	Ocellar Tract D3^3^	*Periplaneta americana*
14	Descending contralateral motion detector^12^,^13^,^89^	*Schistocerca gregaria*
15	DBNc2-1^5^	*Gryllus bimaculatus*
16	Polarization sensitive ipsi DN^11^	*Schistocerca gregaria*
17	DNColHS^90^	*Calliphora erythrocephala, Musca domestica*
18	Giant Antennal Mechanosensory DN^41^	*Drosophila melanogaster*
19	pIP10^15^	*Drosophila melanogaster*
20	Ocellar Tract D2/DCO^3^,^18^,^91^	*Periplaneta americana*
21	DBNc4-1^4^,^92^	*Gryllus bimaculatus*
22	DBNc4-2^4^	*Gryllus bimaculatus*
23	Moonwalking DN^14^	*Drosophila melanogaster*
24	DNOVS1^90^,^93^,^94^	*Calliphora erythrocephala, Musca domestica, Sarcophaga bullata*
25	DNOVS2^90^,^94^,^95^	*Calliphora erythrocephala, Musca domestica, Sarcophaga bullata*
26	Giant Fibre^41^	*Drosophila melanogaster*
GNG		
27	DMIb-1^1^,^96^	*Periplaneta americana*
28	SOG-dc1^4^	*Gryllus bimaculatus*
29	SOG-dc2^4^	*Gryllus bimaculatus*
30	Polarization sensitive SOG DN^11^	*Schistocerca gregaria*
31	OA-VUMd1^55^	*Drosophila melanogaster*
32	OA-VUMd2^55^	*Drosophila melanogaster*
33	OA-VUMd3^55^	*Drosophila melanogaster*
34	DA-DN^52^	*Drosophila melanogaster*
