# Supplementary material for: Organization of descending neurons in Drosophila melanogaster
Source: Sci Rep. 2016 Feb 3;6:20259. doi: 10.1038/srep20259 (PMC4738306; doi:10.1038/srep20259)
Supplement: Supplementary Information [file srep20259-s1.pdf]

Supplementary information.

Organization of descending neurons in *Drosophila melanogaster*.

Authors: Cynthia T. Hsu[1,2] &Vikas Bhandawat[1,2,3]*.

[1]Department of Biology, Duke University.
[2]Deparment of Neurobiology, Duke University.
[3]Duke Institute for Brain Sciences, Duke University.
*Correspondence to: Vikas Bhandawat.
Email: vb37@duke.edu
Phone: 919-684-1703.

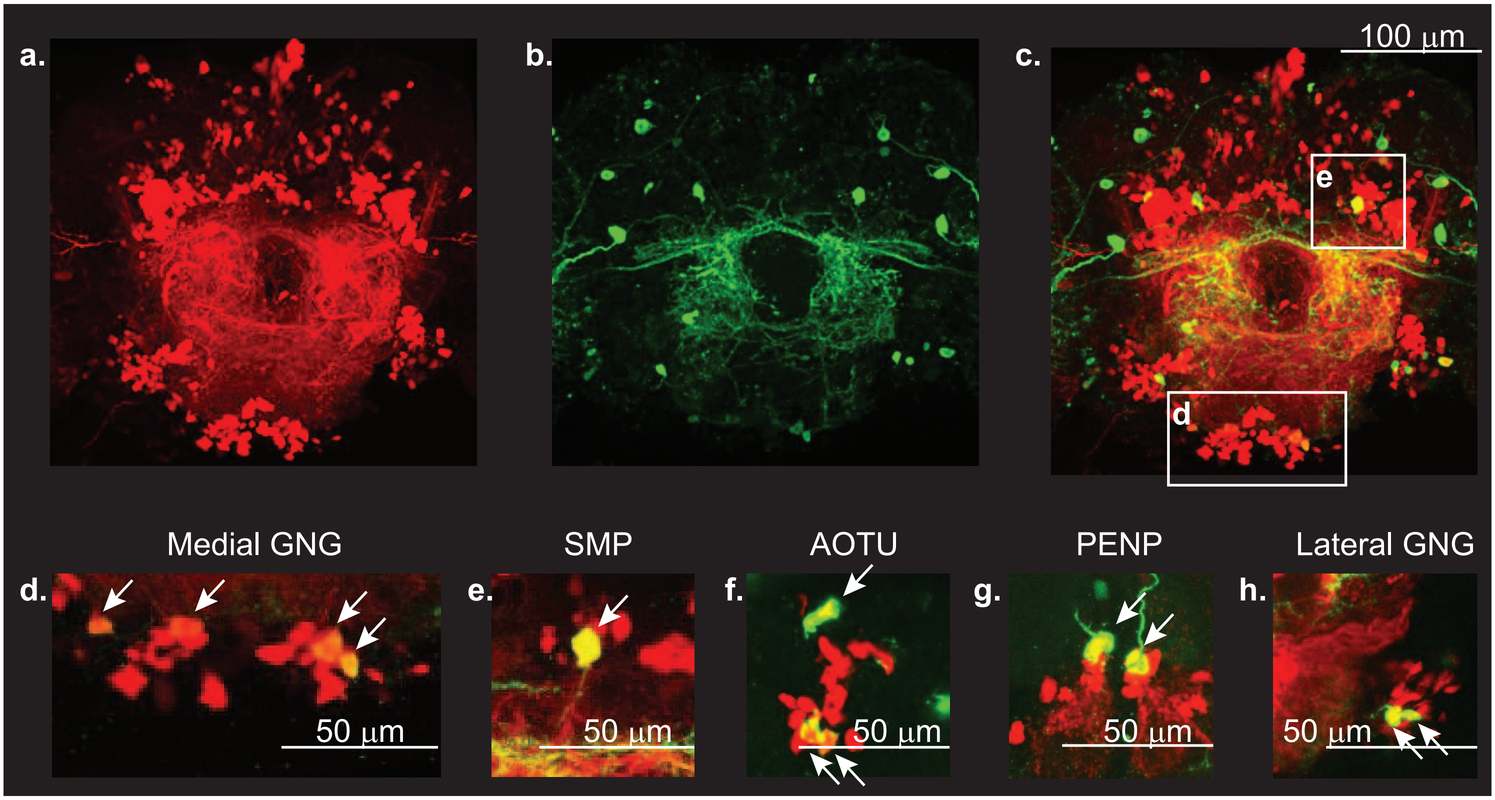

Supplementary Figure S1: Using a sparse genetic label to estimate the efficiency of labeling. E49-Gal4/tsh-Gal80;UAS-CD8GFP labels a small number of DNs which are distributed across five of the six clusters. **(a)** Retrograde labeling to identify the DNs in the brain. **(b)** GFP+ neurons labeled by the E49-Gal4 driver. **(c)** Merge showing colabeling **(d)** Inset showing the GNG cluster. Arrows indicate colabeling (GFP+ DNs). **(e)** Inset showing the colabeled DNs in the SMP cluster. **(f)**, **(g)**, and **(h)** are from a different brain.

We used retrograde labeling in this genotype in two ways. First,we assessed the efficiency of our labeling The number of co-labeled cell bodies in the thoracic ganglia and the brain should closely match the number of axons passing through the cervical connective. The maximum number of axons in the neck connective out of the 5 we measured is 18. The maximum number of GFP+ cell bodies we labeled was 29, 18 of which were in the brain and 11 in the thoracic ganglia. We interpret this result to reflect the fact that the number of axons counted in the neck connective is an underestimate. Although this experiment did not allow us to quantiatively estimate the labeling efficiency, the simplest interpretation is that the labeling efficiency is high.

Second, because we know that there are exactly 18 DNs labeled by E49-Gal4, it allows us to improve our estimate of the number of DNs in the following way: We arrived at 18 by counting the maximum number of DNs in each cluster out of the many bulk-labeling experiments we performed; the maximum number of neurons in any given brain was 15. Thus, the best labeling efficiency for DNs labeled by E49-Gal4 was 83%. If we assume that this efficiency is representative of the overall labeling efficiency, then the number of DNs labeled in this brain - 882.5 - represents 83% of all DNs. Therefore, we estimate the actual number of DNs as 1060.

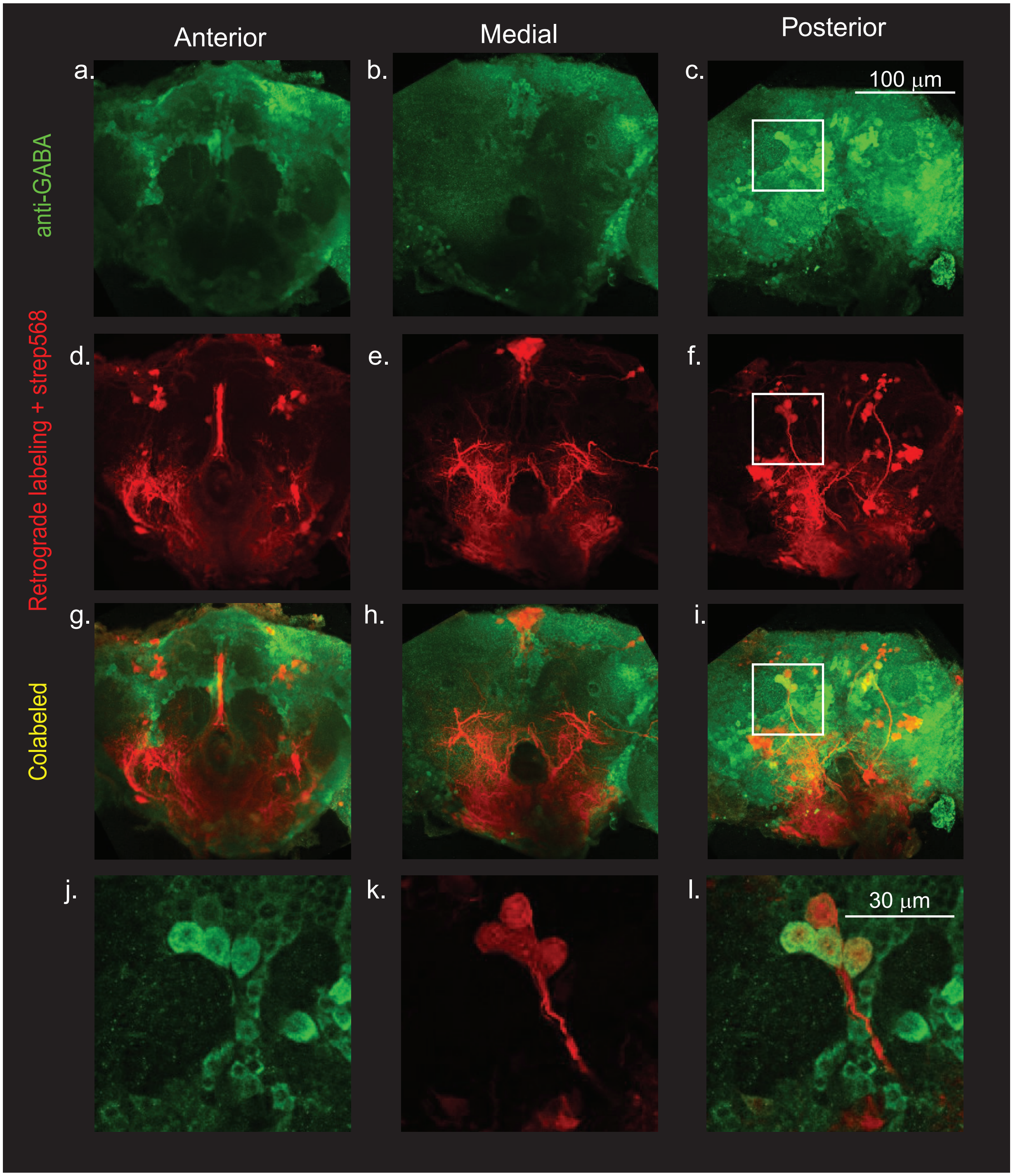

Supplementary Figure S2: GABAergic DNs identified using colabeling of retrograde labeling and GABA antibody. (a-i) Projection of a confocal stack of a retrogradely labeled brain in which all GABAergic neurons are labeled (green). Retrograde label is in red. GABAergic DNs are colabled and appear yellow. **(j-l)** Inset shows a close-up of the region in white square.

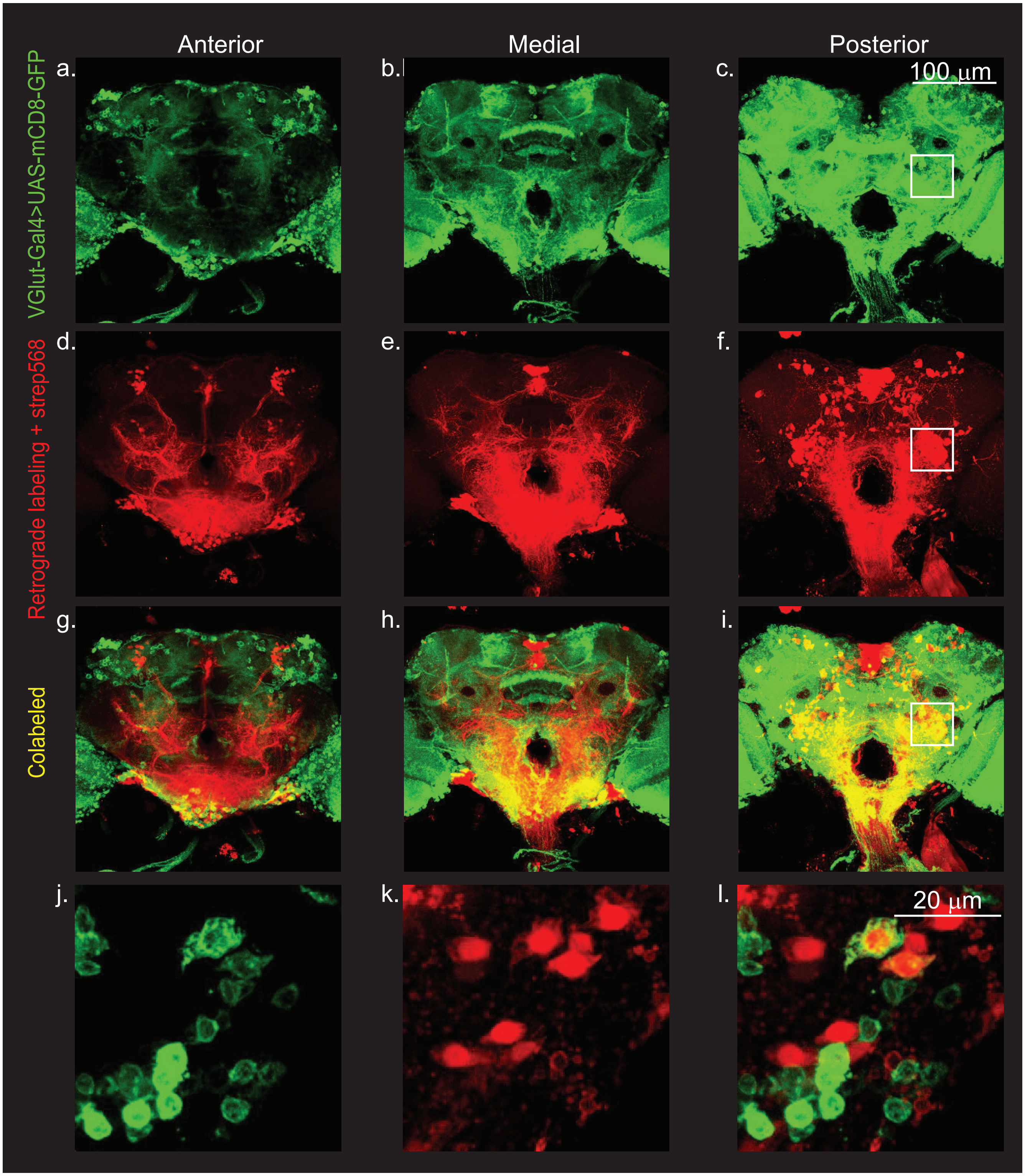

Supplementary Figure S3: Retrograde labeling in flies in which a Gal4 driver specific to glutamatergic neurons, VGlut, was used to drive expression of a reporter gene. (a-i) Projection of a confocal stack of a retrogradely labeled brain in which all glutamatergic neurons are labeled (green). Retrograde label is in red. Glutamatergic DNs are colabeled and appear yellow. **(j-l)** Close-up of the region in white square show the co-labeled neurons.

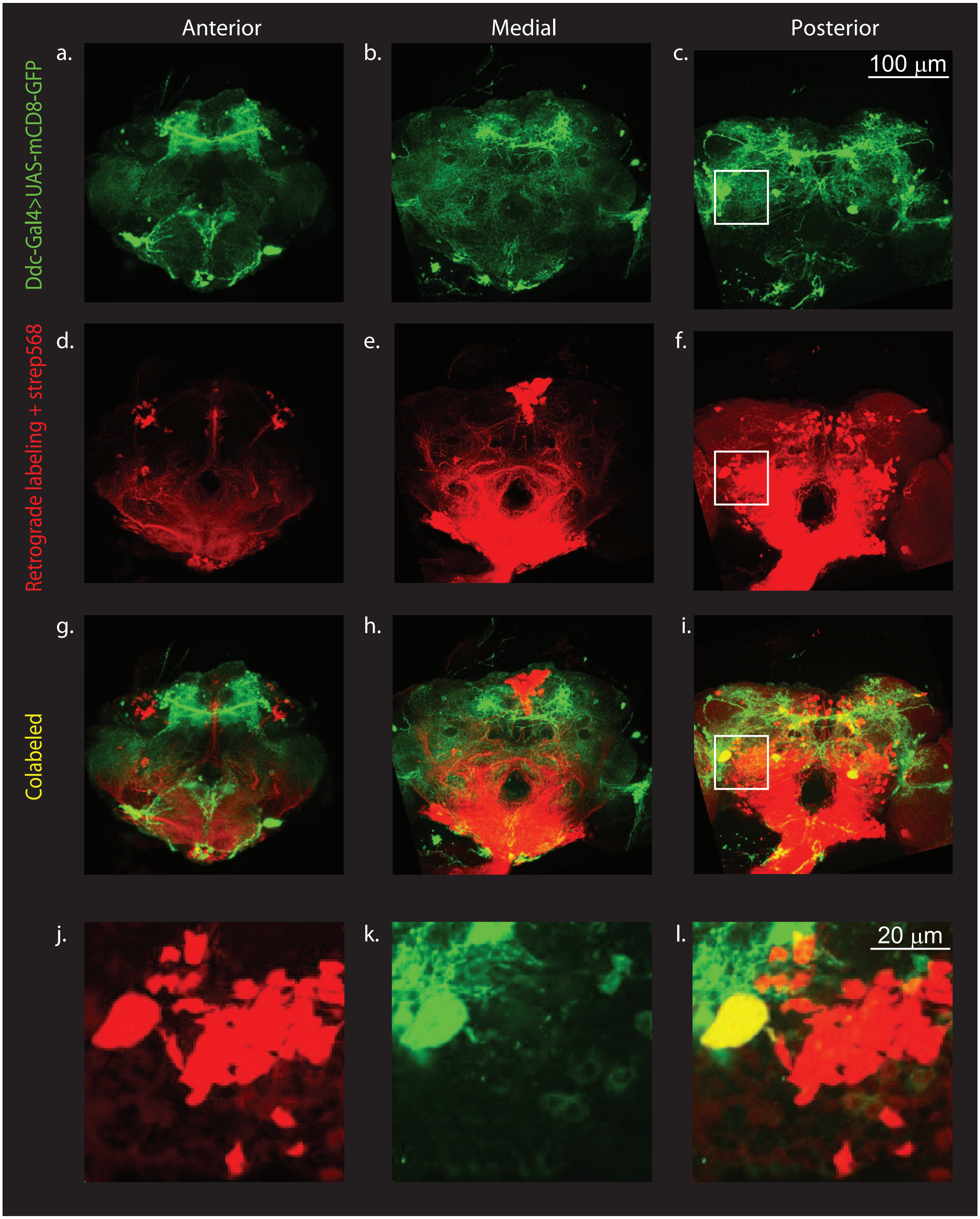

Supplementary Figure S4: Retrograde labeling in flies in which a Gal4 driver specific to serotonergic and dopaminergic neurons, Ddc, was used to drive expression of the reporter gene, UAS-mCD8-GFP. (a-i) Projection of a confocal stack of a retrogradely labeled brain in which all serotonergic neurons and dopaminergic neurons are labeled (green). Retrograde label is in red. Serotonergic and dopaminergic DNs are colabled and appear yellow. **(j-l)** Close-up of the region in white square show the co-labeled neurons.

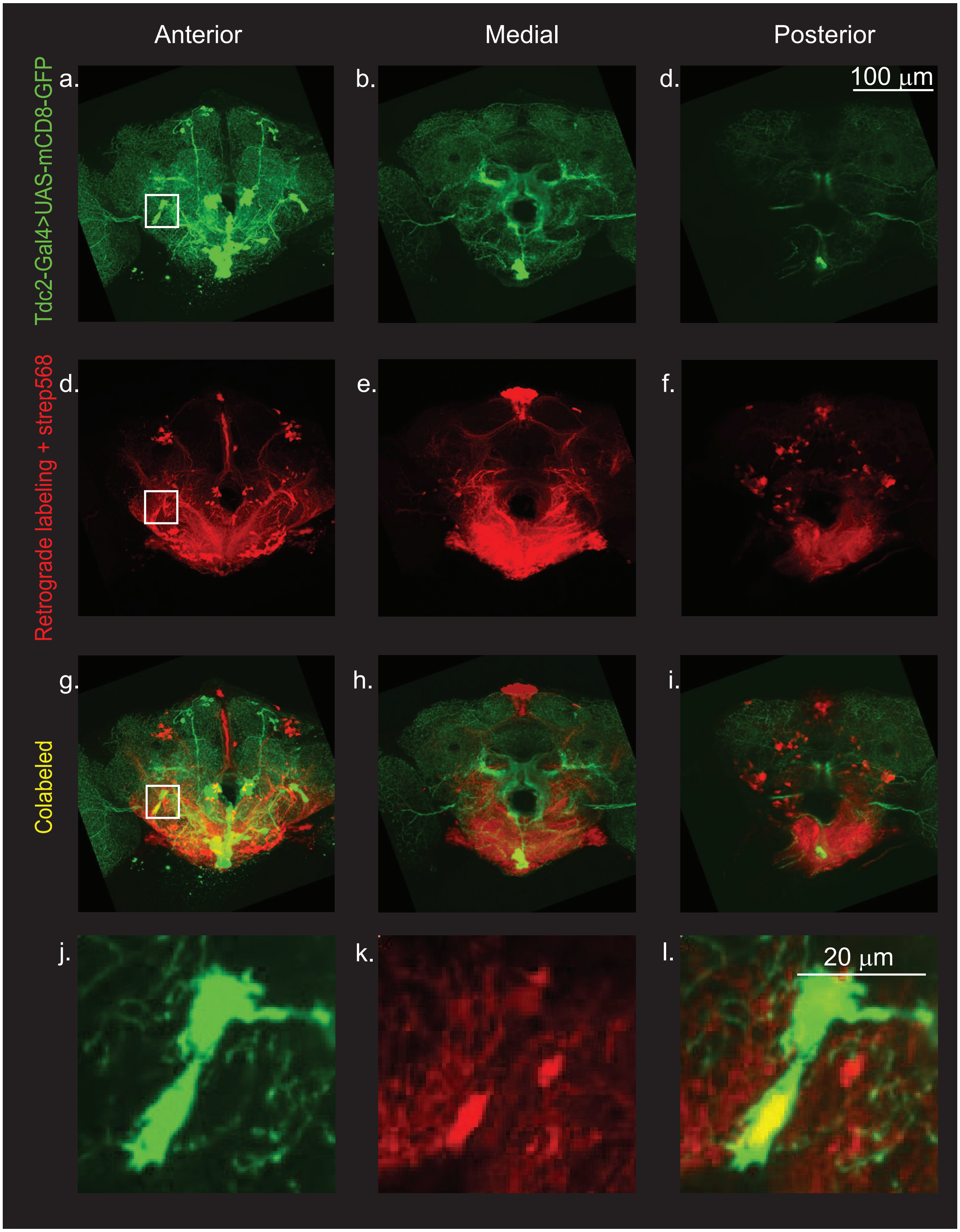

Figure S5: Retrograde labeling experiments were repeated in flies in which a Gal4 driver specific to octopaminergic neurons, Tdc2, was used to drive expression of a reporter. (a-i) Projection of a confocal stack of a retrogradely labeled brain in which all octopaminergic neurons are labeled (green). Retrograde label is in red. Octopaminergic DNs are colabeled and appear yellow. **(j-l)** Inset shows a close-up of the region in white square.

Supplementary Table S1: Neurotransmitter Specific DNs by Cluster

Tdc2-Gal4

Cluster Name	Mean ±SD	Median	Max
AOTU (n=8)	0	0	0
AVLP (n=8)	0.86 ±1.1	0	2 (per hemisphere)
PENP (n=4)	1.5 ±1.7	1.5	3
PI (n=4)	0	0	0
SMP (n=4)	0	0	0
Medial GNG (n=4)	4±1.4	4.5	5
Lateral GNG (n = 8)	0	0	0
Total (n=4)	6.5±1.3	7.5	9

*For AOTU and avPB clusters, the n is listed as the number of hemispheres, rather than brains (n=10 instead of n=5).

Ddc-Gal4

Cluster Name	Mean ±SD	Median	Max
AOTU (n=8)	0	0	0
AVLP (n=8)	0	0	0
PENP (n=8)	0	0	0
PI (n=4)	0.5±0.6	0.5	1
SMP (n=8)*	4.5±2.4	4	9 (per hemisphere)
Medial GNG (n=4)	3.25±1.5	3	5
Lateral GNG (n=8)*	2±1.3	2	4
Total	16.8±2.4	16.5	20

VGlut-Gal4

Cluster Name	Mean ±SD	Median	Max
AOTU (n=6)	0	0	0
AVLP (n=6)	2.2±2.0	2	5 (per hemisphere)
PENP (n=6)	0	0	0
PI (n=3)	2±1	2	3
SMP (n=6)*	4.7±2.3	4	9 (per hemisphere)
Medial GNG (n=3)	14±5.6	13	20
Lateral GNG (n=6)*	4±2.1	3.5	8 (per hemisphere)

w^{1118} colabeled with anti-GABA

Cluster Name	Mean ±SD Fraction Colabeled	Median Fraction Colabeled	Max Cell Bodies Colabeled/Total Bulk Labeled for that Cluster (in an individual fly)	Cell bodies colabeled/Max bulk labeled (in an individual fly)
AOTU (n=6)	0.144 ± 0.078	0.133	4/17	2/20
AVLP (n=6)	0.150 ± 0.168	0.183	¾	2/8
PENP (n=3)	0.182 ± 0.257	0.182	4/11	4/11
PI (n=3)	0.242 ± 0.094	0.231	14/41	14/41
SMP (n=4)	0.468 ± 0.149	0.469	107/165	107/165
Medial GNG (n=3)*	0.309 ± 0.047	0.315	35/99	26/100
Lateral GNG (n=6)*	0.132 ± 0.156	0.136	14/95	14/95

*Delineations between medial and lateral GNGs were estimates.

Cha-Gal4

Cluster Name	Mean ±SD Fraction Colabeled	Median Fraction Colabeled	Max Cell Bodies Colabeled/Total Bulk Labeled for that Cluster (in an individual fly)	Cell bodies colabeled/Max bulk labeled (in an individual fly)
AOTU (n=8)	0.577 ± 0.167	0.577	12/15	12/18
AVLP (n=6)	0.350 ± 0.136	0.303	3/5	3/11
PENP (n=3)	0.645 ± 0.233	0.529	11/12	10/20
PI (n=3)	0.286 ± 0.094	0.313	5/16	4/22
SMP (n=3)	0.246 ± 0.142	0.327	76/231	76/231
Medial GNG (n=3)*	0.404 ± 0.108	0.436	51/180	51/180
Lateral GNG (n=6)*	0.412 ± 0.132	0.400	44/108	38/108

*Delineations between medial and lateral GNGs were estimates.
